# Effects of N95 Masks on Cerebral Oxygen Saturation and End-Tidal Carbon Dioxide Partial Pressure in Healthcare Workers

**DOI:** 10.5812/aapm-135081

**Published:** 2023-11-05

**Authors:** Jahangir Ghorbani, Fatemeh Doraneh-Gard, Seyed Bashir Mirtajani, Mohammad Shirvani, Majid Golestani Eraghi, Seied-Reza Seied-Mohammad Doulabi, Alireza Jahangirifard

**Affiliations:** 1 Chronic Respiratory Diseases Research Center, National Research Institute of Tuberculosis and Lung Diseases, Shahid Beheshti University of Medical Sciences, Tehran, Iran; 2 Working-Group: Immune-Modulation, Department 3 of Medical, University Hospital Munich, Munich, Germany; 3 Lung Transplantation Research Center, National Research Institute of Tuberculosis and Lung Diseases, Shahid Beheshti University of Medical Sciences, Tehran, Iran; 4 Anesthesiology Research Center, Shahid Beheshti University of Medical Sciences, Tehran, Iran; 5 Department of Otolaryngology, Masih Daneshvari Hospital, Tehran, Iran

**Keywords:** N95 Masks, Cerebral Oxygen Saturation, Carbon Dioxide, Healthcare Workers

## Abstract

**Background:**

Healthcare workers must wear masks throughout their shifts, especially those in operating rooms for long periods.

**Objectives:**

This study evaluated the effects of wearing N95 masks on blood and cerebral oxygen saturation levels for three hours.

**Methods:**

The present case-control study enrolled 20 operating room workers wearing N95 masks. Their blood oxygen saturation (SaO_2_), end-tidal carbon dioxide partial pressure (P_ET_CO_2_), and right- and left-sided cerebral regional oxygen saturation (rSO_2_) were measured in the beginning (0 h) and after wearing N95 masks for one and three hours.

**Results:**

Wearing a mask affected P_ET_CO_2_, rSO_2_, and pulse rate and caused fatigue and lightheadedness in some cases. The participants' mean P_ET_CO_2_ increased significantly, from 32 mmHg before putting on a mask to 38 mmHg after wearing it for three hours (P < 0.05). No significant change was observed in the participants' mean rSO_2_, though changes in their rSO_2_ levels were recorded (P > 0.05).

**Conclusions:**

We showed evidence of changes in different physiology parameters due to using masks for 1 to 3 h. Notably, wearing an N95 mask increased end-tidal carbon dioxide partial pressure and decreased cerebral oxygen saturation in individual cases, not all cases.

## 1. Background

Facemasks are a critical component of personal protective equipment, especially for healthcare workers. When such workers faced the spread of the novel pathogen SARS-CoV-2 (COVID-19 virus), there was a lack of scientific data regarding the physiological effects of facemasks.

The World Health Organization (WHO) proposed the extensive use of facemasks by individuals experiencing symptoms of COVID-19 and the general use of facemasks in, for example, crowded places. It particularly makes sense for healthcare workers who come into direct contact with patients and work for long periods in enclosed environments (1-3). However, uncertainties remain regarding how best to use surgical masks and ventilators to protect healthcare workers treating COVID-19 patients, PTS, etc. Several healthcare organizations have published studies on whether and to what extent surgical masks offer sufficient protection from COVID-19 compared to N95 masks. The studies (4) indicate that N95 masks, which filter out approximately 95% of small particulate substances from the air (including the COVID-19 virus), make breathing difficult for the person wearing the mask. These masks could reduce oxygen intake by 5 - 20%, which may be an important reduction rate (5). There are several warning signs of reduced oxygen intake, including vertigo and lightheadedness; such symptoms affect the performance of healthcare workers, particularly those who must wear masks for many hours while working in clinics (6).

Measuring cerebral oxygenation constitutes a noninvasive technique that allows for the constant assessment of tissue oxygenation. Cerebral oximeters can determine cerebral regional oxygen saturation (rSO_2_) in the brain and represent an index of cerebral oxygenation. The supply of oxygen to the brain can be affected by various physiological variables, including blood oxygenation, cardiac output, pulmonary function, blood pressure, hemoglobin, and vasoactive, all involved in regulating cerebral oxygenation. Modifying most of these physiological processes changes an individual's rSO_2_ (7). Both cerebral and pulse oximeters use light transmission and absorption to quantify the ratio of oxygenated to deoxygenated hemoglobin in cerebral tissue (2). However, there is a difference between these two kinds of oximetry (8), as cerebral oximetry seems to be more sensitive and accurate than pulse oximetry (9). The measurement of tissue oxygen saturation by cerebral oximetry reflects the saturation of hemoglobin in cerebral tissue (10) and the equilibrium between oxygen supply and demand. Pulse oximetry measures an individual's oxygen supply rather than the delivery of oxygen (11, 12).

Assessing cerebral oxygenation limits postoperative injury and mortality disruption by indicating problematic oxygenation at the systemic and tissue levels (13). Maintaining continuous information related to cerebral blood and oxygen is vital, as it can offer increased protection (14).

It has been hypothesized that the use of masks may cause hypoxia and affect cerebral oxygenation. The present study investigated whether cerebral oxygen saturation, as measured in hemoglobin and end-tidal carbon dioxide partial pressure (P_ET_CO_2_), was affected by wearing an N95 mask.

Careful peril-profit analyses of the potential long-term effects of masks are becoming increasingly important for patients and healthcare professionals. Although studies have been done on pregnant healthcare workers, the data on all healthcare workers during the coronavirus pandemic are unavailable.

## 2. Objectives

This study aimed to provide an initial, rapid, and scientific presentation of the risks and potential adverse medical effects of masks, particularly among specific diagnostic groups, patients, and other user groups.

## 3. Methods

The present case-control study was conducted over 3 months (July 1, 2020, to October 1, 2020) at Masih Daneshvari Hospital, Shahid Beheshti University of Medical Sciences, Tehran, Iran. We examined 20 healthy medical personnel working in operating rooms (age 35 ± 8.3 years), including 66% males and 33% females. They were included in the study if they were under 65 years old and willing to follow the study protocol. Exclusion criteria were cerebrovascular diseases, convulsions, respiratory disease (pulmonary or inflammatory), ischemic heart disease, and uncontrolled blood hypertension or diabetes. We excluded workers with a baseline O_2_ saturation of less than 94% while breathing room air and who could not continue wearing the mask during the study. The study procedures were explained to all participants before the experiment started, and all participants were required to sign voluntary written forms, as mandated by the Declaration of Masih Daneshvari Hospital.

### 3.1. Procedure

Capnography, pulse oximeter, and cerebral oximetry were used to measure and record the participants' relative levels of excreted carbon dioxide (CO_2_), oxygen saturation (SaO_2_) of arterial pulsation, and rSO_2_. Their physiological parameters, including right-sided and left-sided rSO_2_, P_ET_CO_2_, SaO_2_, pulse rate (PR), respiratory rate (RR), and mean arterial pressure (MAP) were measured before (time zero, baseline) and after they wore N95 masks during their routine working day in an operating room. As mentioned above, all physiological parameters were measured again after one and 3 hours of continuous mask-wearing. Correct connections and leak tightness were confirmed during the experiment. The present study defined physiological parameters as a negative effect of masks as a relevant measure.

### 3.2. Statistical Analysis

We used SPSS v. 21.0 (IBM, Armonk, NY, United States) to analyze the data. Mean ± standard deviations are provided. Statistical comparisons of the two groups were made using a two-tailed *t*-test (if the data was normally distributed) and the Mann-Whitney Wilcoxon test (if the data was not normally distributed). Statistical analyses were performed with Microsoft Excel 2010^®^ (Microsoft, Redmond, Washington, USA). Differences were considered as 'not significant' with P values > 0.1, 'tendentially significant' (significant*) with P values between 0.1 and 0.05, 'significant' (significant**) with P values between 0.05 and 0.005, and 'highly significant' (significant***) with P values < 0.005. Correlations were defined as "negligible" with r values of 0.00 to 0.30 (-0.00 to -0.30), "low" with r values of 0.30 to 0.50 (-0.30 to -0.50), moderate" with r values of 0.50 to 0.70 (-0.50 to -0.70), and "high" with r values of 0.70 to 1.00 (-0.70 to -1.00). Figures were created with GraphPad Prism7^©^ (GraphPad Software, California, USA).

## 4. Results

Results are given at three distinct time points for all 20 healthy volunteers. Physiological parameters of P_ET_CO_2_, SaO_2_, cerebral oxygenic right-sided and left-sided (rSO_2_), PR, and RR were measured three times before (baseline) and after (60 min and 180 min) wearing N95 facemasks.

### 4.1. P_ET_CO_2_ and SaO_2_

The results showed a significant change in P_ET_CO_2_ concentrations, though this effect was not seen with SaO_2_. When participants wore masks, we found a combined effect of N95 respiratory protection and carbon dioxide rise. Nevertheless, we did not find a similar result for the decrease in oxygen saturation.

In addition, we addressed whether CO_2_ is increased due to mask-wearing. According to data, mask wearers showed outstanding frequency changes due to mask-wearing.

Mean P_ET_CO_2_ at baseline was 32 (26.0 - 36.0) mmHg for non-mask-wearers, which rose to 35 (28.0 - 41.0) mmHg after 1 h and 38 (30.0 - 44.0) mmHg after 3 h of wearing an N95 mask. Therefore, we had a statistically significant increase (P < 0.05) in blood CO_2_ after continuous mask-wearing.

**Figure 1.**
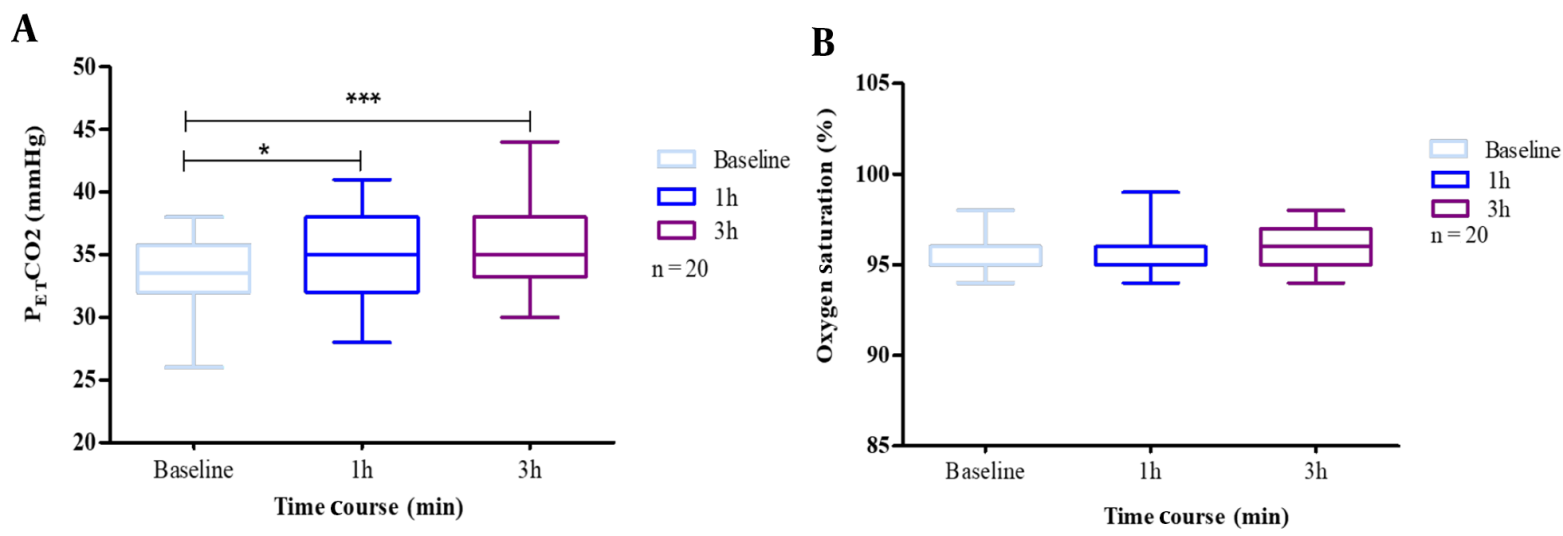
A, end-tidal carbon dioxide partial pressure (P_ET_CO_2_); B, oxygen saturation (SaO_2_), at baseline, 1 h after, 2.5 h after. The exercise test was performed by 20 subjects with N95 masks. Box plots represent the changes in the oxygen saturation and carbon dioxide due to mask use. The results are presented as cases that used masks. The whiskers represent the local maximum and minimum values; each box's horizontal line represents the median. Differences were considered 'tendentially significant' (*) with P-values of 0.1 - 0.05 and 'highly significant' (***) with P-values < 0.005.

Baseline SaO_2_ was 96.0% (95.0% - 98.0%). The effect of masks was not statistically significant regarding blood oxygen saturation (SpO_2_) after the first, second, and third hours. The analysis results of SaO_2_ and P_ET_CO_2_ are provided in Figure 1. 

### 4.2. Lightheadedness and Fatigue

**Figure 2.**
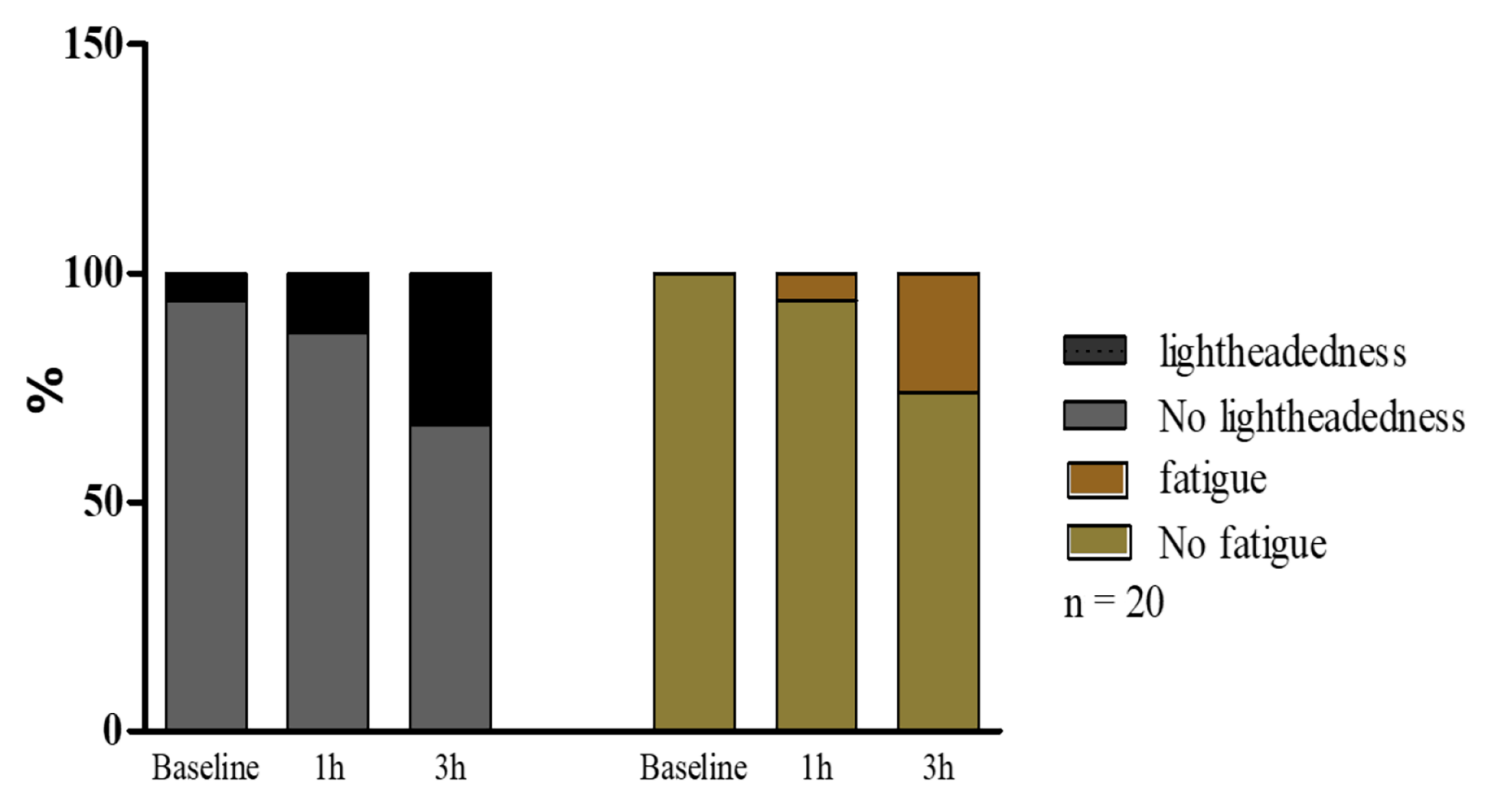
The occurrence of lightheadedness and fatigue before wearing a mask (baseline) and after 1 h and 3 h of wearing a mask. Lightheadedness and fatigue were increased after 1 h and particularly after 3 h. Nevertheless, it was insignificant in healthcare workers compared to the baseline after mask-wearing.

The study revealed that 25% (5 out of 20) of volunteers experienced lightheadedness. Furthermore, oxygen deprivation due to N95 respiratory masks was associated with fatigue in 20% (4 out of 20) of cases after 2.5 h of mask-wearing compared to the baseline. The dual occurrence of the physical parameters of lightheadedness and fatigue was found in 20% of the participants (Figure 2). 

### 4.3. PR, RR, and MAP

Our results indicated that N95 facemasks can induce different effects on pulse rate (heart rate) at distinct time points. However, baseline PR while not wearing a mask was 82.0 (62.0 - 107.0) bpm vs. 83.0 (74.0 - 113.0) bpm after 1 h and 75.0 (57.0 - 96.0) bpm after 3 h, which represent a predictable increase in RR 1 h after mask-wearing. On the other hand, PR decreased to an amount lower than the baseline after a longer duration of mask-wearing.

Furthermore, the baseline RR was 18 (10.0 - 26.0) rpm vs. 18.0 (10.0 - 27.0) rpm after 1 h and 17.0 (8.0 - 25.0) rpm after 3 h. The results showed a decrease in RR, but not significantly after 3 h compared to the baseline.

**Figure 3.**
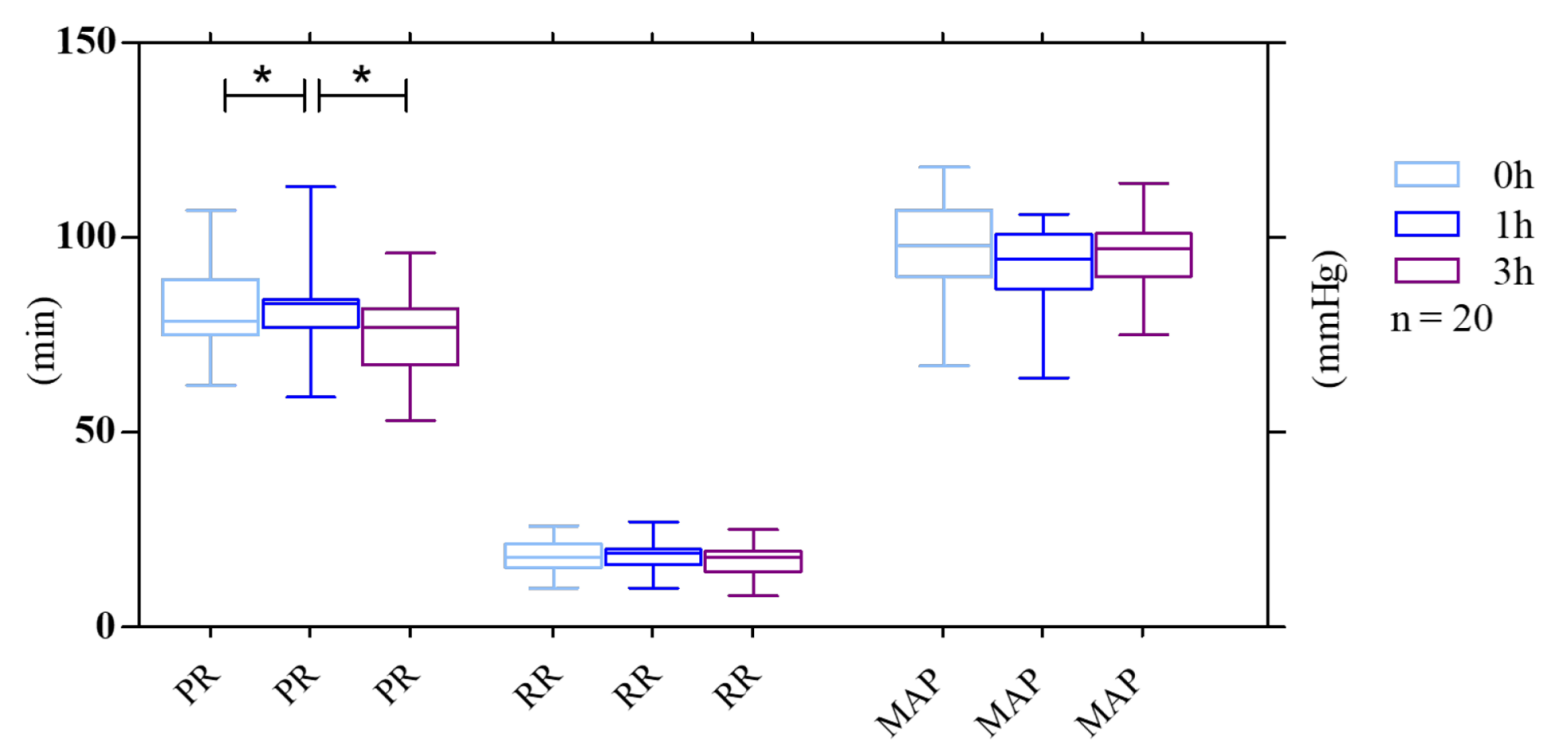
Mean changes in physiological parameters throughout the exercise test were performed by 20 subjects with surgical masks and N95 respirators. Pulse rate (PR, pulsations/min), respiratory rate (RR, breaths/min), and mean arterial pressure (MAP) at the three-time points were considered. The whiskers represent the local maximum and minimum values; each box's horizontal line represents the median. Differences were considered 'tendentially significant' (*) with P-values of 0.1 - 0.05.

Concerning MAP, the mean baseline was 96.0 (67.0 - 118.0) mmHg vs. 93.0 (78.0 - 106.0) mmHg after 1 h and 96.0 (75.0 - 114.0) mmHg after 3 h. After using masks for 1 h, we observed a reduction in blood pressure that was not statistically significant. It did not continue for the next 2 h and returned to the normal baseline measurement (Figure 3). 

### 4.4. Cerebral Regional Oxygen Saturation

**Figure 4.**
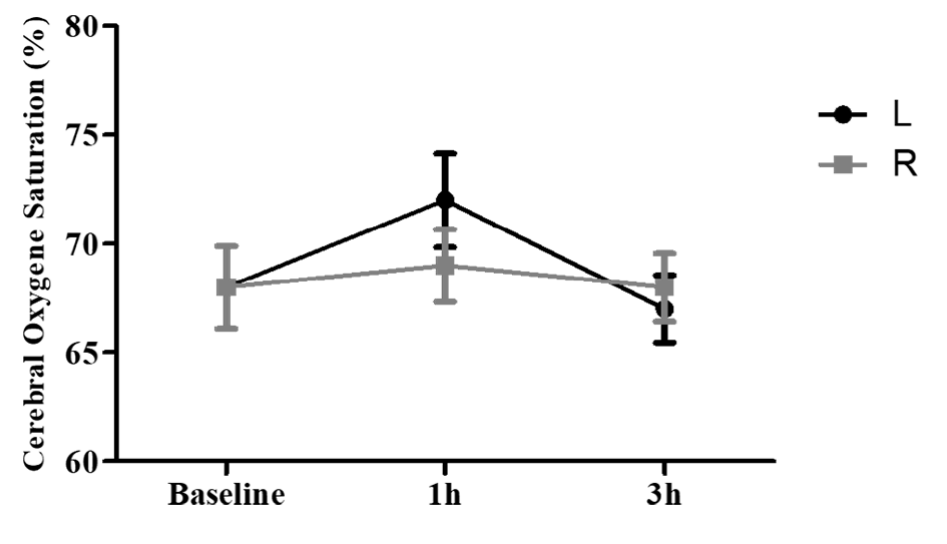
Relative changes in cerebral regional oxygen saturation (rSO_2_) for all subjects after using a mask compared to baseline (0 h). Data are expressed as mean ± S.E.M.

The mean rSO_2_ for the right and left parts of the brain following 3 h of using a mask showed no significant change (more than 20%) compared to the baseline (0 h) (Figure 4). The mean rSO_2_ right side at baseline was 68% (55.0 - 82.0), which rose to 69% (55.0 - 92.0) after 1 h and decreased to 68% (53.0 - 79.0) after 3 h of mask-wearing. Regarding the left side of rSO_2_, the mean value at baseline was 68% (59.0 - 80.0), which rose to 72% (52.0 - 94.0) after 1 h and decreased to 67% (50.0 - 78.0) after 3 h of mask-wearing. An increase in rSO_2_ (less than 20%) on the right side after 3 h compared with the baseline (0 h) was evident for some other individuals. Measurement of rSO_2_ on the right side for a volunteer at baseline was 84%, which rose to 92% after 1 h and decreased to 79% after 3 h of mask-wearing. Further, a few subjects who experienced a remarkable change showed a decrease in rSO_2_ (more than 20%) in the left side of the brain compared to the baseline.

**Figure 5.**
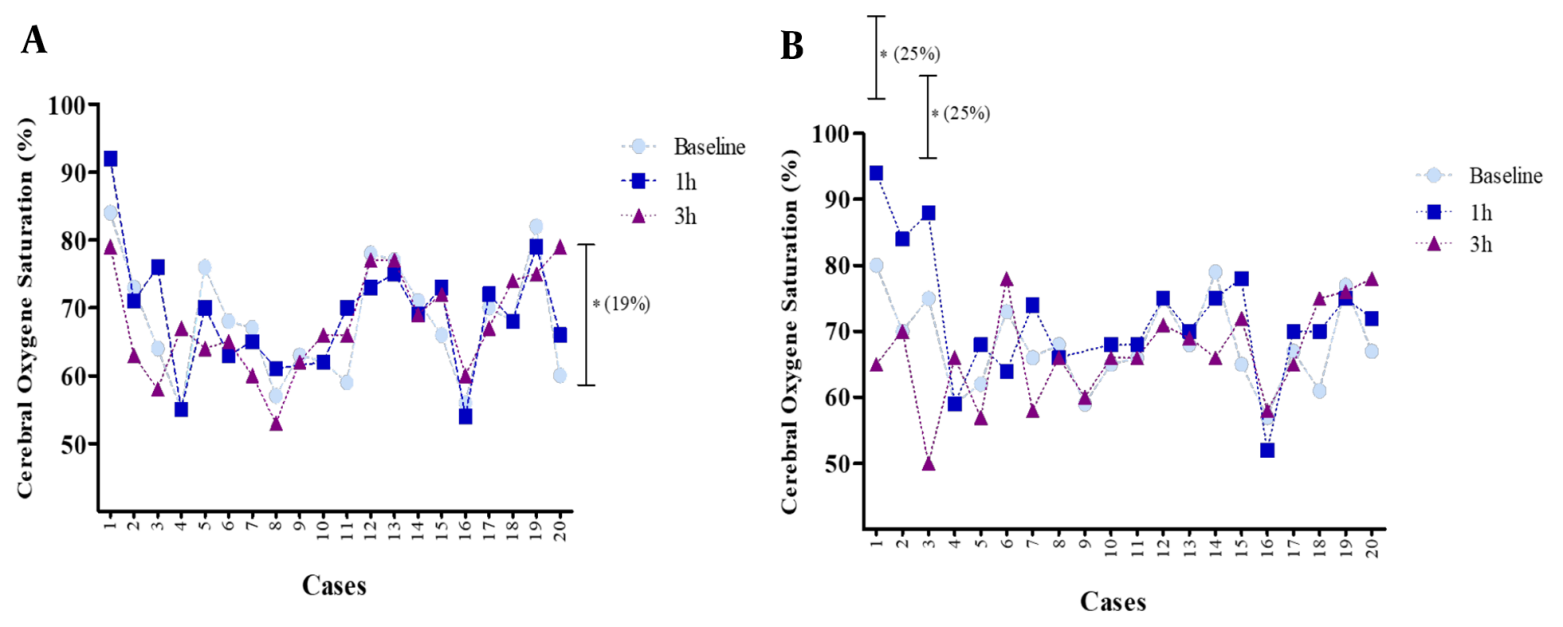
Relative changes in cerebral regional oxygen saturation (rSO_2_) on the right (A), and left (B) sides after using a mask compared to baseline (0 h) for each subject. Data are expressed as mean ± S.E.M. for each of the 20 cases * significant differences (more than 20% from baseline). Each dot indicates the oxyhemoglobin saturation in first‐degree and second‐degree venules at a specific point in time.

The measurements of left-side rSO_2_ for three volunteers at baseline were 80%, 75%, and 66%, respectively, which rose to 94%, 88%, and 74% after 1 h. Furthermore, these values decreased to 65%, 50%, and 58% after 3 h of mask-wearing (Figure 5). 

### 4.5. Correlation of Lightheadedness with P_ET_CO_2_ and rSO_2_

**Figure 6.**
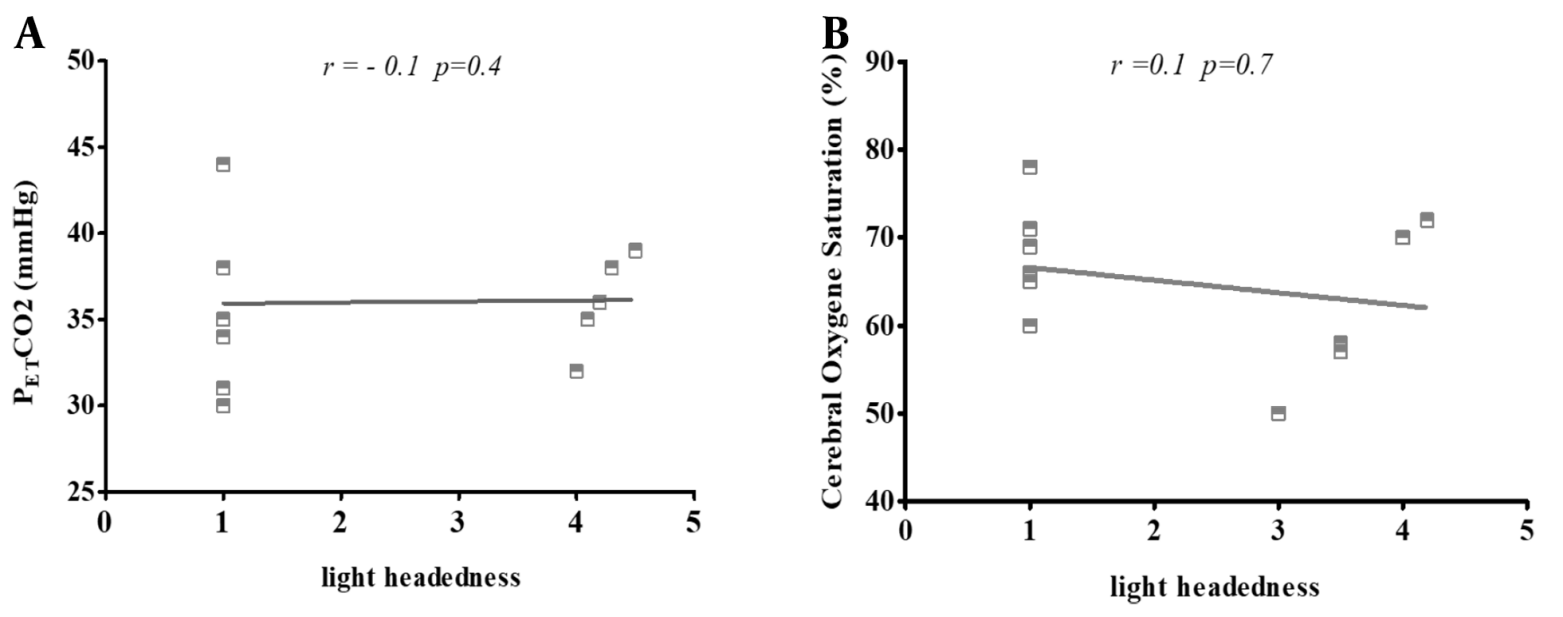
Pearson's correlation scatters plot of lightheadedness with (A) end-tidal carbon dioxide partial pressure (P_ET_CO_2_); and (B) cerebral oxygen saturation (rSaO_2_) in 20 subjects with N95 masks. The results present cases in which the use of masks exhibited no significant correlation with lightheadedness and two other variants. The correlation was defined as "negligible" with r values of 0.00 to 0.30 (-0.00 to -0.30), "low" with r values of 0.30 to 0.50 (-0.30 to -0.50), "moderate" with r values of 0.50 to 0.70 (-0.50 to -0.70), and "high" with r values of 0.70 to 1.00 (-0.70 to -1.00).

We observed two statistically significant changes in physiological parameters during the study, both in P_ET_CO_2_ and rSO_2_. Therefore, we did not have a statistically significant correlation in the quantitative analysis between P_ET_CO_2_ and rSO_2_ and headache/fatigue as some accompanying factors among mask wearers (Figure 6). 

## 5. Discussion

The permanent use of N95s can influence cerebral oxygen, pulse rate, respiratory rate, and oxygen saturation, among others. The primary argument of this study is that masks influence the oxygen level of the brain and carbon dioxide levels. In contrast, blood oxygen saturation does not show any change. Previous studies have shown that N95 masks reduce the oxygen concentration of blood by more than 20 - 25%. Therefore, it is vital to thoroughly assess the risk factors of wearing facemasks for long durations (15, 16). It is also vital to have further studies to find an alternative for surgeons who frequently have long procedures in operation rooms. Wearing a mask can be associated with certain physiological parameters. However, no significant differences were found for all parameters (16-18).

Our results show that the use of a mask by healthcare workers does not have large and statistically consistent effects on key physiological parameters, such as oxygen saturation and respiratory rate, but does indeed affect pulse rate, carbon dioxide partial pressure, and cerebral oxygen. In the present study, health workers in operating rooms were impressed while wearing N95 masks at different time points. In addition, we observed increases in P_ET_CO_2_ associated with mask-wearing. A small but significant increase in P_ET_CO_2_ showed a clearer effect in our study, possibly due to CO_2_ rebreathing among the staff wearing masks. As in other studies, there were a few subjects with lung or heart diseases connected to P_ET_CO_2_, which should be considered when wearing masks (19).

An increase in P_ET_CO_2_ was also found with face masks used routinely (20). In another study, using N95 masks increased carbon dioxide tension/partial pressure (PCO_2_) among lung-healthy users but without a major physiologic burden (20, 21).

Trouble breathing with masks can be associated with certain neurological responses, such as increased impulses from the highly thermosensitive area of the face covered by the mask or temperature of the circulating air and an increase in P_ET_CO_2_ (22). Our findings demonstrate that using a mask during aerobic training has minimal and statistically inconsistent effects on major physiological parameters such as HR, RR, BP, and SO_2_. Moreover, cerebral oximetry was reduced by 5 - 20% in our volunteers wearing masks. Values of rSO_2_ are affected by other physiologic variables that determine brain oxygen supply and demand. Alterations in most of these variables result in a symmetrical effect on rSO_2_ (13).

In order to avoid hypoxia in the brain, a sufficient source of oxygen is critical. The amount of rSO_2_ levels is sensitive to changes in both arterial oxygen and carbon dioxide blood content (23). Maintaining adequate oxygen delivery to tissues and organs, particularly the brain, is of fundamental importance. The dangers of prolonged hypoxia and reduced oxygen delivery to the brain are well documented (24, 25).

The parameters of right-sided and left-sided rSO_2_ showed different patterns. In the first hour of mask-wearing, we saw an increase in right and left-side rSO_2_ of the brain; however, the increase in rSO_2_ on the left side was higher compared to the baseline, which was more than the amount of rSO_2_ on the right side of the brain. Normally, differences of up to 10% in cerebral oxygenation between the left and right hemispheres are apparent, especially during unstable arterial saturations, which may indicate uneven cerebral oxygenation (1).

Increases in P_ET_CO_2_ after 1 h can be associated with increased cerebral oxygen in the brain's left and right sides. One reason may be that cerebrovascular response to the hypoxic condition is caused by increased P_ET_CO_2_ after mask-wearing. Therefore, P_ET_CO_2_ can be used as the stimulus for increased cerebral oxygen. The increase was insignificant; after 3 h, cerebral oxygen decreased to values even less than the bassline (26). The effects of oxygen deficiency overcome vasodilation due to increased carbon dioxide, and we observed a decrease in rSO_2_ after a 3 h exposure to the hypoxic situation (mask). Another reason could be more oxygen consumption over time, followed by more mental work than the baseline, which can cause a decrease in rSO_2_. Earlier studies demonstrated that rSO_2_ is affected by changes in cardiac output and oxygen consumption. However, it seems to be independent of MAP (as cerebral oxygen saturation), which changes in arterial oxygen content (i.e., hypoxemia, anemia) and could have an impact on cardiac outcomes and corresponding rSO_2_ (27).

It is known that cerebral metabolic rate is related to oxygen delivery. Due to decreased arterial oxygen, we will have decreased oxygen delivery. In this case, this cerebral autoregulation mechanism maintains cerebral oxygen delivery via proportionate increases in cerebral blood flow.

In the case of steady arterial oxygen content, decreases in rSO_2_ imitate an increased oxygen extraction ratio. A reduction in cerebral oximetry values > 20% from baseline has been identified as a reliable, sensitive, and specific threshold for detecting brain ischemia (28).

In previous studies focusing on healthy people, surgical face masks were associated with an increased heart rate (29). Our results demonstrate that wearing masks in two ways can influence pulse rate. After just one hour of mask-wearing, we saw an increase in pulse rate. We observed a decrease compared to the baseline after three hours of mask-wearing. An increase in PR might result from an O_2_ decrease in tissue in the form of demand of the body to compensate for reducing oxygen via cardiac output, causing an increase in PR. As the body wants to compensate for the decrease of oxygen via cardiac output, PR will increase (30). Increased activity of breathing or respiratory muscle to increase cardiac output can cause an increase in PR (2, 29).

Furthermore, as inducing cardiac output cause of hypoxia condition is restricted in both diastolic and systolic diameters, we had a decrease in PR after 3 h of mask-wearing (31). On the other hand, vasodilation could cause an increase in arterial blood flow and help decrease arterial heart rate and blood pressure (27).

Concerning MAP, we saw an increase after one hour of mask-wearing and a decrease after three hours. Normally, based on a previous study, the pulse rate increase is associated with higher blood pressure. This could have the same template and explanation for MAP with PR (32).

One possible explanation for participants who experienced headaches can be the compression of sensitive facial skin and superficial nerves by the face mask and its tight straps, especially when worn longer (33).

Based on the previous study, rSO_2_ and P_ET_CO_2_ can contribute to fatigue and lightheadedness. The correlation of lightheadedness with P_ET_CO_2_ and rSO_2_ in the present study was insignificant, although a correlation existed. This may be due to the number of studied cases, which were insufficient for correlation analysis (33, 34).

There were a few limitations in the present study that may complicate our results:

1. Certain brain areas remained unmonitored as cerebral oximeters only measure regional cerebral oxygen supply.

2. The effect of N95 masks was tested on only a few persons, which could be a reason for significant differences after wearing the mask compared to the baseline value. Other factors like BMI should also be addressed. Moreover, the physiological effect of mask-wearing can vary in different situations with different physical actions.

3. The duration of mask-wearing should be extended and not restricted to just three points in time. Further time points would provide more insight into the effect of mask-wearing on cerebral oxygen levels or oxygen saturation.

### 5.1. Conclusions

The purpose of wearing facemasks is to protect the wearers against viruses. It is important to determine whether masks can provide sufficient protection for healthcare workers while affecting various physiological parameters, particularly cerebral oxygen saturation, carbon dioxide partial pressure, PR, and MAP.

## References

[1] Lemmers PM, van Bel F (2009). Left-to-right differences of regional cerebral oxygen saturation and oxygen extraction in preterm infants during the first days of life.. Pediatr Res..

[2] Li Y, Tokura H, Guo YP, Wong AS, Wong T, Chung J (2005). Effects of wearing N95 and surgical facemasks on heart rate, thermal stress and subjective sensations.. Int Arch Occup Environ Health..

[3] Sommerstein R, Fux CA, Vuichard-Gysin D, Abbas M, Marschall J, Balmelli C (2020). Risk of SARS-CoV-2 transmission by aerosols, the rational use of masks, and protection of healthcare workers from COVID-19.. Antimicrob Resist Infect Control..

[4] Khurana S, Soda N, Shiddiky MJA, Nayak R, Bose S (2021). Current and future strategies for diagnostic and management of obstructive sleep apnea.. Expert Rev Mol Diagn..

[5] Tong PS, Kale AS, Ng K, Loke AP, Choolani MA, Lim CL (2015). Respiratory consequences of N95-type Mask usage in pregnant healthcare workers-a controlled clinical study.. Antimicrob Resist Infect Control..

[6] Sjaastad O, de Souza Carvalho D, Fragoso YD, Zhao JM (1988). Cluster headache: on the significance of so-called minibouts.. Cephalalgia..

[7] Fikenzer S, Uhe T, Lavall D, Rudolph U, Falz R, Busse M (2020). Effects of surgical and FFP2/N95 face masks on cardiopulmonary exercise capacity.. Clin Res Cardiol..

[8] Vegh T (2016). Cerebral Oximetry in General Anaesthesia.. Turk J Anaesthesiol Reanim..

[9] Tosh W, Patteril M (2016). Cerebral oximetry.. BJA Educ..

[10] Kaya A, Okur M, Sal E, Peker E, Kostu M, Tuncer O (2014). Comparison of Cerebral Oximeter and Pulse Oximeter Values in the First 72 Hours in Premature, Asphyctic and Healthy Newborns.. West Indian Med J..

[11] Fulesdi B, Limburg M, Bereczki D, Kaplar M, Molnar C, Kappelmayer J (1999). Cerebrovascular reactivity and reserve capacity in type II diabetes mellitus.. J Diabetes Complications..

[12] Settakis G, Pall D, Molnar C, Bereczki D, Csiba L, Fulesdi B (2003). Cerebrovascular reactivity in hypertensive and healthy adolescents: TCD with vasodilatory challenge.. J Neuroimaging..

[13] Vretzakis G, Georgopoulou S, Stamoulis K, Stamatiou G, Tsakiridis K, Zarogoulidis P (2014). Cerebral oximetry in cardiac anesthesia.. J Thorac Dis..

[14] Farzanegan B, Eraghi MG, Abdollahi S, Ghorbani J, Khalili A, Moshari R (2018). Evaluation of cerebral oxygen saturation during hypotensive anesthesia in functional endoscopic sinus surgery.. J Anaesthesiol Clin Pharmacol..

[15] Akgül MŞ, Ozcan N, Uzun ME, Gurses VV, Baydil B (2021). Physiological impact of wearing a surgical face mask during walking in the COVID-19 pandemic.. Pedagogy Phys Cult Sports..

[16] Chen Y, Yang Z, Wang J, Gong H (2016). Physiological and subjective responses to breathing resistance of N95 filtering facepiece respirators in still-sitting and walking.. Int J Ind Ergon..

[17] Kyung SY, Kim Y, Hwang H, Park JW, Jeong SH (2020). Risks of N95 Face Mask Use in Subjects With COPD.. Respir Care..

[18] Person E, Lemercier C, Royer A, Reychler G (2018). [Effect of a surgical mask on six minute walking distance].. Rev Mal Respir..

[19] Epstein D, Korytny A, Isenberg Y, Marcusohn E, Zukermann R, Bishop B (2021). Return to training in the COVID-19 era: The physiological effects of face masks during exercise.. Scand J Med Sci Sports..

[20] Rhee MSM, Lindquist CD, Silvestrini MT, Chan AC, Ong JJY, Sharma VK (2021). Carbon dioxide increases with face masks but remains below short-term NIOSH limits.. BMC Infect Dis..

[21] Roberge RJ, Coca A, Williams WJ, Powell JB, Palmiero AJ (2010). Physiological impact of the N95 filtering facepiece respirator on healthcare workers.. Respir Care..

[22] Samannan R, Holt G, Calderon-Candelario R, Mirsaeidi M, Campos M (2021). Effect of Face Masks on Gas Exchange in Healthy Persons and Patients with Chronic Obstructive Pulmonary Disease.. Ann Am Thorac Soc..

[23] Strapazzon G, Gatterer H, Falla M, Dal Cappello T, Malacrida S, Turner R (2021). Hypoxia and hypercapnia effects on cerebral oxygen saturation in avalanche burial: A pilot human experimental study.. Resuscitation..

[24] Murkin JM (2011). Cerebral oximetry: monitoring the brain as the index organ.. Anesthesiology..

[25] Murkin JM, Arango M (2009). Near-infrared spectroscopy as an index of brain and tissue oxygenation.. Br J Anaesth..

[26] Fierstra J, Sobczyk O, Battisti-Charbonney A, Mandell DM, Poublanc J, Crawley AP (2013). Measuring cerebrovascular reactivity: what stimulus to use?. J Physiol..

[27] Siddiqui A (2011). Effects of Vasodilation and Arterial Resistance on Cardiac Output.. J Clin Exp Cardiol..

[28] Moritz S, Kasprzak P, Arlt M, Taeger K, Metz C (2007). Accuracy of cerebral monitoring in detecting cerebral ischemia during carotid endarterectomy: a comparison of transcranial Doppler sonography, near-infrared spectroscopy, stump pressure, and somatosensory evoked potentials.. Anesthesiology..

[29] Lassing J, Falz R, Pokel C, Fikenzer S, Laufs U, Schulze A (2020). Effects of surgical face masks on cardiopulmonary parameters during steady state exercise.. Sci Rep..

[30] Hong C, Olsen BD, Hammond PT (2022). A review of treatments for non-compressible torso hemorrhage (NCTH) and internal bleeding.. Biomaterials..

[31] Zarndt R, Piloto S, Powell FL, Haddad GG, Bodmer R, Ocorr K (2015). Cardiac responses to hypoxia and reoxygenation in Drosophila.. Am J Physiol Regul Integr Comp Physiol..

[32] Reule S, Drawz PE (2012). Heart rate and blood pressure: any possible implications for management of hypertension?. Curr Hypertens Rep..

[33] Nybo L, Rasmussen P (2007). Inadequate cerebral oxygen delivery and central fatigue during strenuous exercise.. Exerc Sport Sci Rev..

[34] Uygun O, Ertas M, Ekizoglu E, Bolay H, Ozge A, Kocasoy Orhan E (2020). Headache characteristics in COVID-19 pandemic-a survey study.. J Headache Pain..

